# Data on maltreatment profiles and psychopathology in children and adolescents

**DOI:** 10.1016/j.dib.2016.07.056

**Published:** 2016-08-06

**Authors:** Andreas Witt, Annika Münzer, Helene G. Ganser, Jörg M. Fegert, Lutz Goldbeck, Paul L. Plener

**Affiliations:** University of Ulm, Department of Child and Adolescent Psychiatry/Psychotherapy, Germany

**Keywords:** Child maltreatment, Latent class analysis, Child abuse and neglect, Psychopathology

## Abstract

We present data on maltreatment profiles and psychopathology of 358 children and adolescents (4–17 years). Data on maltreatment profiles has been categorized into six major maltreatment types: physical abuse, emotional abuse, sexual abuse, sexual abuse with penetration, exposure to intimate partner violence and neglect. The data on history of maltreatment is based on the interview version of the Juvenile Victimization Questionnaire (JVQ). Additionally data on psychopathology in general as well as specific disorders according to DSM-IV based on K-SADS-PL is presented. The data was used to examine patterns of co-occurrences of maltreatment and associated clinical outcome variables using latent class analysis (LCA), “Experience by children and adolescents of more than one type of maltreatment: association of different classes of maltreatment profiles with clinical outcome variables” (Witt et al.,) [1].

**Specifications Table**

**Table:** 

Subject area	*Psychology*
More specific subject area	*Child Maltreatment*
Type of data	*Table, Excel file*
How data was acquired	*Survey, Structural Equation Modeling, Latent class analysis*
Data format	*Raw, analyzed*
Experimental factors	*Participants underwent assessments for history of maltreatment and psychopathology using clinical interviews and completed standardized questionnaires. On basis of the clinical interviews, participants were categorized whether they had experienced different maltreatment type to retrieve maltreatment profiles. Latent class analysis was used to identify latent classes of maltreatment profiles*
Experimental features	*Clinical assessment with standardized clinical interviews and questionnaires*
Data source location	*Ulm, Datteln and Lüneburg, Germany*
Data accessibility	*Data is within this article*

**Value of the data**

• These data can be used in larger analyses on the psychological outcomes of child maltreatment and to examine differential effects of different types of maltreatment. These data characterize individual patterns of maltreatment history across 6 major types of maltreatment: physical abuse, emotional abuse, sexual abuse, sexual abuse with penetration, exposure to domestic violence, neglect and psychopathology that can be used to compare.
• The data provide useful information on the co-occurrence of different maltreatment types in a large clinical sample.
• The data could be helpful in analyses of co-occurrences of different types of child maltreatment.
• The data could be used in meta-analysis on the impact of different types of child maltreatment on outcome variables.

## 1 Data

The data (Supplmentary Table 1) includes data on the maltreatment profiles and psychopathology of children and adolescents, derived from the CANMANAG study.^1^ They include:

– Sociodemographic data (age and sex).
– Type of maltreatment (physical abuse, emotional abuse, sexual abuse, sexual abuse with penetration, exposure to domestic violence and neglect)
– Data on psychopathology (overall diagnosis according to DSM-IV, specific diagnosis of ADHD, Conduct Disorder, Oppositional Defiant Disorder, Mood Disorder, Excretion Disorder and Level of Functioning).
– Class membership based on the latent class analysis (LCA).
– Class probabilities based on the LCA (Table 1).

## 2 Experimental design, materials and methods

### 2.1 Design and procedure

The data was collected as part of the CANMANAGE program. Data were collected between 2012 and 2015 at three study centers in Germany. Assent from participants and written informed consent from legal guardians were obtained before any assessments were performed. The study was approved by the local institutional review board at each center (Application #122/12). Participants received an incentive of 20€ for taking part.

Children and adolescents with a known history of maltreatment who were clients of child welfare institutions or of mental health services were referred to one of the study centers. The history of maltreatment was known to the accompanying caregivers and the staff of local child welfare and medical services. Potential participants were contacted by local coordinators, informed about the study, and invited to enroll. Those who agreed to participate underwent clinical assessments and completed a set of standardized questionnaires. The Juvenile Victimization Questionnaire (JVQ) was administered to the participants and their caregivers. The participants were informed about the background, content and course of the assessment. Additionally participants and caregivers were informed that there was the possibility to provide further information in the course of the assessment when caregivers and participants were interviewed separately. After the JVQ, psychopathology was assessed using the German version of the Schedule of Affective Disorders and Schizophrenia for School-Age Children (K-SADS). For children aged eight years and older, the child and the caregivers were interviewed separately. For children younger than eight years only the primary caregiver was interviewed. The children could choose whether they wished to stay with their caregiver or play in a separate room. This procedure was taken to ensure quality and completeness of the data.

### 2.2 Sociodemographics

The 358 participants consisted of 202 males, and 156 females. The age range in the sample was 4–17 years, with a mean of 10.18 years (*SD*=3.41). With respect to place of residence, 209 (58.4%) were living with at least one parent, 120 (33.5%) were in an out-of-home placement, and 29 (8.1%) had different living arrangements, such as living alone or other relatives. For 183 participants (51.1%), the primary caregiver was the mother, for 31 (8.7%) it was the father and for 15 (4.2%) it was mother and father. For 12 (3.4%) the primary caregivers were relatives other than the parents (e.g. grandparents) and for 46 (12.8%) it was foster parents. For another 71 (19.8%) the primary caregiver was another person, in most cases a professional social worker.

### 2.3 Maltreatment profiles

Child abuse and neglect profiles were assessed using the German adaptation of the Juvenile Victimization Questionnaire (JVQ) [2], [3]. The child and the caregiver were assessed together. When the child was 8 years or older, predominantly the child was addressed and the caregivers could add important information. When the child was younger than 8 years, also the child was addressed, but the caregiver was the main source of information. The 24-item version assesses lifetime exposure, and includes only items from modules B (child maltreatment), D (sexual victimization), and E (witnessing and indirect victimization). On basis of their answers (yes/no) to the 24 items of the JVQ, participants were categorized whether they had experienced one of the six major maltreatment types: physical abuse, emotional abuse, sexual abuse, sexual abuse with penetration, exposure to domestic violence and/or neglect. The six major maltreatment types are represented by one or more of the 24 items of the JVQ.

### 2.4 Psychopathology

Psychopathology was assessed using the German version of the Schedule of Affective Disorders and Schizophrenia for School-Age Children (K-SADS-PL) [4]. The instrument was used to determine the presence of any diagnosis according to DSM-IV and the presence of 6 specific disorders: Attention Deficit Hyperactivity Disorder (ADHD), Conduct Disorder, Oppositional Defiant Disorder, Posttraumatic Stress Disorder (PTSD), Mood Disorders and Excretion Disorders. Interviews were conducted by trained and supervised interviewers who held degrees at the master׳s level. To ensure quality, the first two assessments conducted by each interviewer were videotaped, and feedback was provided. For participants aged eight years and older, the child and the primary caregiver were interviewed separately; for children younger than eight, only the caregiver was interviewed.

### 2.5 Level of Functioning

To assess the level of psychosocial functioning of children on a continuous scale that ranges from 1 to 100, the Children׳s Global Assessment Scale (C-GAS) [5]. After the full assessment, the level of psychosocial functioning of the participant was rated by the interviewer.

### 2.6 Latent class analysis

The classes were calculated on basis of the maltreatment profiles that were obtained on basis of the JVQ using MPlus version 7.0 [6]. In an explorative approach, estimates for the number of classes were calculated. The best fitting model contained three classes. Model fit parameters are presented in Witt et al. [1]. As an indicator of reliability of the solution, the class probabilities are also presented.

## Figures and Tables

**Table 1:** Included measures.

*Data categories*	*Specific measures*	*Labels/Values*
*Sociodemographic*	*ID*	
	*Age*	*In years*
	*Sex*	1=*male*, 2=*female*
*Child maltreatment profiles*	*Physical abuse*	0=*no,* 1=*yes*
	*Emotional abuse*	0=*no,* 1=*yes*
	*Sexual abuse*	0=*no,* 1=*yes*
	*Sexual abuse with penetration*	0=*no,* 1=*yes*
	*Exposure to domestic violence*	0=*no,* 1=*yes*
	*Neglect*	0=*no,* 1=*yes*
*Psychopathology*	*Diagnosis according to DSM-IV*	0=*no,* 1=*yes*
	*ADHD*	0=*no,* 1=*yes*
	*Conduct Disorder*	0=*no,* 1=*yes*
	*Oppositional Defiant Disorder*	0=*no,* 1=*yes*
	*PTSD*	0=*no,* 1=*yes*
	*Mood Disorder*	0=*no,* 1=*yes*
	*Excretion Disorder*	0=*no,* 1=*yes*
*Level of Functioning*	*CGAS*	1–100
*Latent class analysis*	*LCA*	1=*Class* 1, 2=*Class* 2, 3=*Class* 3
	*Cluster* 1 (*Class Probability Class* 1)	0–1
	*Cluster* 2 (*Class Probability Class* 2)	0–1
	*Cluster* 3 (*Class Probability Class* 3)	0–1

## Footnotes

1 www.canmanage.de.
