# Supplementary material for: Data on maltreatment profiles and psychopathology in children and adolescents
Source: Data Brief. 2016 Aug 6;8:1352–6. doi: 10.1016/j.dib.2016.07.056 (PMC4993853; doi:10.1016/j.dib.2016.07.056)
Supplement: Supplementary file 1 — Supplementary material [file mmc1.docx]

Declaration of Conflicts of Interests

Within the last five years PLP received funding for a clinical study by Lundbeck Pharamceuticals. Furthermore he received research funding from the Federal Agency of drugs and medical products (BfArM), the German Federal Ministry of Education and Research (BMBF) and the Baden-Wuerttemberg Ministry of Research and Art. He received funding from the state foundation of Baden-Wuerttemberg, as well as from the foundation for outpatient child and adolescent psychiatry. He declares no conflict of interest with regards to this publication

Within the last five years JMF received research funding from the European Union (EU), German Research Foundation (DFG), the German Federal Ministry of Health (BMG), the German Federal Ministry of Education and Research (BMBF), the German Federal Ministry of Family Affairs, Senior Citizens, Women and Youth (BMFSFJ), several state ministries of social affairs, State Foundation Baden-Württemberg, Volkswagen Foundation, European Academy, Pontifical Gregorian University, RAZ, CJD, Caritas, Diocese of Rottenburg-Stuttgart. He received travel grants, honoraria and sponsoring for conferences and medical educational purposes from the German Research Foundation (DFG), the American Academy of Child and Adolescent Psychiatry (AACAP), the National Institute of (Mental) Health (NIMH/NIH), the European Union (EU), Pro Helvetica, Janssen-Cilag (J&J), Shire, several universities, professional associations and German federal and state ministries. He conducted clinical trials for Janssen-Cilag, Lundbeck, the German Federal Ministry of Education and Research (BMBF) and Servier. He is in steering committees and DSMB for Lundbeck, Servier. Every grant and honorarium has to be declared to the law office of the University hospital Ulm. Potential conflicts of interests have to be declared to German Society for Child and Adolescent Psychiatry and Psychotherapy (DGKJP) and American Academy of Child and Adolescent Psychiatry (AACAP) annually, because of commission membership. He has no stocks, no interests in pharmaceutical companies and is majority owner of the 3Li institute.

The other author(s) declared no potential conflict of interest with respect to the research, authorship, and/or publication of this article.

Funding

This analyses is based on data derived from the CANMANAGE study, which is funded by the German Federal Ministry of Education and Research (funding code 01KR1202A). The views expressed in this article are the views of the author(s) and may not necessarily reflect the views of the German Federal Ministry of Education and Research.
